# Clinics and genetic background of hereditary gingival fibromatosis

**DOI:** 10.1186/s13023-021-02104-9

**Published:** 2021-11-24

**Authors:** Karolina Strzelec, Agata Dziedzic, Katarzyna Łazarz-Bartyzel, Aleksander M. Grabiec, Ewa Gutmajster, Tomasz Kaczmarzyk, Paweł Plakwicz, Katarzyna Gawron

**Affiliations:** 1 Department of Molecular Biology and Genetics, Faculty of Medical Sciences in Katowice, Medical University of Silesia, Medykow 18, 40-752 Katowice, Poland; 2 Department of Periodontology and Oral Medicine, Medical College, Jagiellonian University, Kraków, Poland; 3 Department of Microbiology, Faculty of Biochemistry, Biophysics and Biotechnology, Jagiellonian University, Kraków, Poland; 4 Department of Oral Surgery, Medical College, Jagiellonian University, Kraków, Poland; 5 Department of Periodontology and Oral Diseases, Faculty of Dentistry, Medical University of Warsaw, Warsaw, Poland

**Keywords:** Chromosome, Gene, Hereditary gingival fibromatosis, Linkage analysis, Pathogenic variant, Whole-exome sequencing

## Abstract

**Background:**

Hereditary gingival fibromatosis (HGF) is a rare condition characterized by slowly progressive overgrowth of the gingiva. The severity of overgrowth may differ from mild causing phonetic and masticatory issues, to severe resulting in diastemas or malposition of teeth. Both, autosomal-dominant and autosomal-recessive forms of HGF are described. The aim of this review is a clinical overview, as well as a summary and discussion of the involvement of candidate chromosomal regions, pathogenic variants of genes, and candidate genes in the pathogenesis of HGF. The loci related to non-syndromic HGF have been identified on chromosome 2 (GINGF, GINGF3), chromosome 5 (GINGF2), chromosome 11 (GINGF4), and 4 (GINGF5). Of these loci, pathogenic variants of the *SOS-1* and *REST* genes inducing HGF have been identified in the GINGF and the GINGF5, respectively. Furthermore, among the top 10 clusters of genes ranked by enrichment score, ATP binding, and fibronectin encoding genes were proposed as related to HGF.

**Conclusion:**

The analysis of clinical reports as well as translational genetic studies published since the late’90s indicate the clinical and genetic heterogeneity of non-syndromic HGF and point out the importance of genetic studies and bioinformatics of more numerous unrelated families to identify novel pathogenic variants potentially inducing HGF. This strategy will help to unravel the molecular  mechanisms as well as uncover specific targets for novel and less invasive therapies of this rare, orphan condition.

## Background

Gingival fibromatosis (GF), also known as gingival hyperplasia or gingival overgrowth may be manifested as pathologic, diffuse, or local growth of the gingiva. By definition, it affects the masticatory mucosa (the marginal and attached gingiva and the interdental papilla), but it does not spread beyond the mucogingival junction. The excess gingival tissue can cover part of or the entire crown of a tooth, and can result in diastemas, teeth displacement, retention of primary or permanent teeth, and may also cause masticatory, phonetic, psychological, and esthetic problems [1, 2]. In rare cases, the condition may be associated with hereditary factors and occurs as an isolated (non-syndromic) hereditary gingival fibromatosis (HGF, MIM 135300), otherwise known as hereditary gingival overgrowth affecting exclusively gingiva. It can appear as a complete diffuse condition when it affects both the maxilla and the mandible, however, it can also affect exclusively one jaw (part diffuse condition) or it can appear locally as a nodule-like form [3–5]. Both, autosomal-dominant and autosomal-recessive forms of this condition have been reported [3].

The first clinical signs of HGF manifest in the primary dentition period. Parents and/or close relatives usually have had their own experience with this condition and seek medical advice for the child. At this age, the most prominent feature of HGF is an extremely wide zone of keratinized gingiva. During adolescence, the connective tissue within the gingiva appears thickened, mostly due to overproduction of collagenous proteins. Except for recurrent cases, gingival overgrowth in HGF is characterized by a slow progression rate. GF associated with hereditary factors may also co-exist as a part of rare genetic diseases, syndromes, or chromosomal abnormalities, e.g.,Ramon syndrome (MIM 266270), Jones syndrome (MIM 135550), juvenile hyaline fibromatosis (MIM 228600). In the case of genetic syndromes or diseases, gingival overgrowth co-exists with a variety of systemic symptoms, such as skeletal defects, hypertrichosis, epilepsy, sensorineural hearing loss, joint contractures, and others. For more detailed information, refer to Gawron et al. [3].

To date, five distinct loci related to the non-syndromic variant of HGF have been identified, two on chromosome 2 (GINGF on 2p21-p22, MIM 135300 and GINGF3 on 2p22.3-p23.3, MIM 609955) [6–8], one mapped to chromosome 5 (GINGF2 on 5q13-q22, MIM 605544) [9], one to chromosome 11 (GINGF4 on 11p15, MIM 611010) [10], and one to chromosome 4 (GINGF5 on 4q12, MIM 617626) [11]. Of these loci, HGF-inducing pathogenic variants of the *Son-of-Sevenless-1* (*SOS-1*) gene (MIM 182530) and in the *RE1-silencing transcription factor* (*REST)* gene (MIM 600571) have been identified within the GINGF and the GINGF5 loci, respectively. Wild-type human *SOS-1* gene encodes a highly conserved protein, Son-of-Sevenless-1 (SOS-1), and the product of the *REST* gene, is the RE1-silencing transcription factor [11, 12]. Originally, a single-cytosine insertion in the genomic sequence of the *SOS-1* gene (GenBank: NC_000002.12 (NM_005633.4): g.126,142-126,143insC), mapped to the 2p21-p22 locus has been identified as a causative factor of non-syndromic HGF type 1 (HGF1) in one family from Brazil [6, 12]. A dominant HGF locus has been also mapped to the region 2p21-p22 in chromosome 2 in four Chinese families [8]. In contrast to the Brazilian family, where suppression of recombination was found, similar characteristics were not observed in the Chinese families. Furthermore, the *SOS-1* locus was likely not affected in this and one more study of other Chinese families [6, 8, 13]. Similarly, the presence of single-cytosine insertion in the *SOS-1* gene, and the corresponding HGF1 phenotype have not been found in two affected families from the Polish population investigated in our study [14]. Otherwise, whole-exome sequencing (WES) analysis of eleven affected individuals with HGF from three unrelated families, carried out by Bayram et al., led to the identification of three heterozygous pathogenic variants  predicted by conceptual translation to result in premature termination of the nascent transcript, two frameshifts, and one nonsense allele in the *REST* gene [11]. This gene has been mapped to the GINGF5 locus on chromosome 4 (4q12). Likewise, the group from Korea performed WES followed by gene set enrichment analysis (GSEA), and a protein functional network study in two affected donors from a three-generation family and proposed the candidate representative genes of the clusters of related genes potentially associated with non-syndromic HGF. They proposed ATP binding and fibronectin gene clusters as the central candidate that may cause gingival overgrowth [15]. The clinical communications, as well as the reports from translational studies on the genetics of HGF published over the last twenty years, suggest a heterogeneous nature of non-syndromic HGF however, so far, an updated overview of this topic has not been published.

In line with this, the aim of this review is a clinical overview, as well as a summary and discussion of the involvement of candidate chromosomal regions, candidate genes, and pathogenic variants of genes in the pathogenesis of HGF.

## Clinical manifestations  of HGF

**Fig. 1:**
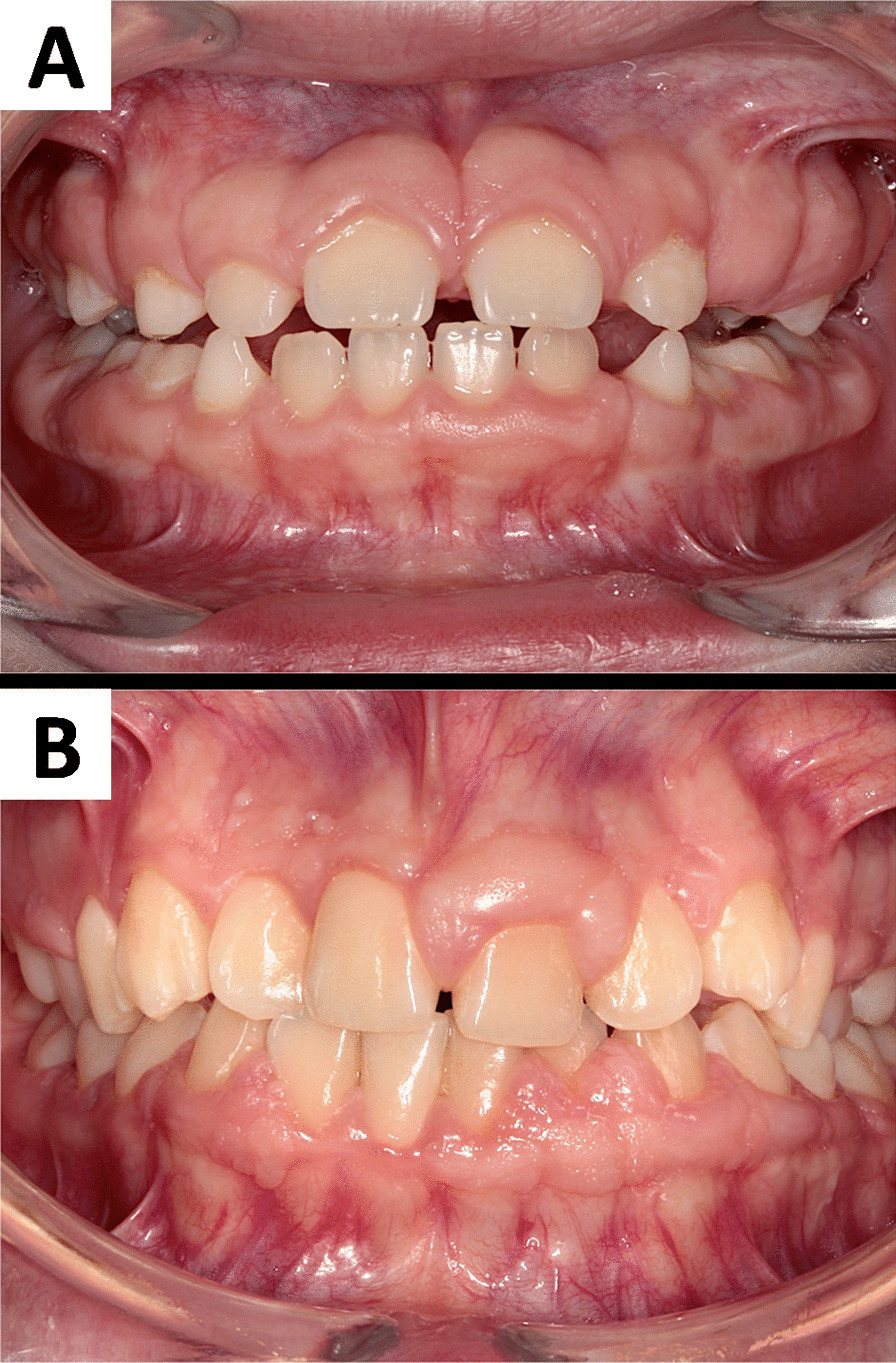
Phenotypic heterogeneity of hereditary gingival fibromatosis. **A.** the photography shows the gingiva from a 10-year-old Caucasian male patient diagnosed with HGF. Both mother of the patient and her sister are also affected. The surface of the gingiva is almost homogeneous, smooth and with a normal stippling of the attached gingiva. An unusually wide zone of the keratinized gingiva is equally distributed along teeth in both dental arches in the maxilla and in the mandible. The margin of gingiva obscures half of the crowns’ height, which makes teeth appearing not completely erupted; **B**.  a 15-year-old Caucasian female diagnosed with asymmetric gingival overgrowth of hereditary origin in the maxilla and in the mandible. Hypertrophy of gingiva caused spacing between teeth. It also makes clinical crowns appearing to be shorter than their anatomical length. Gingiva around some teeth looks almost normal while in other locations it presents a significant amount of keratinized tissue, which covers the teeth surface. The surface of the gingiva is heterogeneous at different sites of oral cavity. Some areas are smooth with a normal stippling of the gingiva, whereas other resemble multiple verrucous lesions. Some interdental papillae are overgrown, inflamed, and bleed easily during tooth brushing

Isolated (non-syndromic) HGF (MIM 135300) is a rare genetic disease with unknown prevalence, manifested by a slowly progressive, non-hemorrhagic, benign, and fibrous overgrowth of the gingiva. The incidence and severity of the disease appear to depend on the penetrance of the mutated gene [6, 7]. Hypertrophic gingiva is usually normal in colour and consists of dense fibrous tissue that feels firm and nodular on palpation. Gingival overgrowth, in addition to being disfiguring, leads to compromised quality of life of the patient due to increased difficulty to maintain oral hygiene, which may lead to caries development, and several periodontal and orthodontic problems [1, 16–18]. Clinically, the onset coincides with the eruption of primary or permanent dentition and is rarely present at birth [19]. Males and females are affected equally. Many reports, including clinical cases or case series present variable clinical aspects of this condition. Whereas the majority of the reports present diffuse (generalized) appearance of gingival overgrowth in both, the mandible and the maxilla or less commonly within only one jaw, others describe exclusively local presence of nodule-like overgrowth around a group of teeth in one or both dental arches [1, 3, 20, 21]. The cases described differ in the severity of the overgrowth. In mild and moderate conditions, phonetic and masticatory problems are usually observed. Severe overgrowth, which covers the major part or entire crowns of the teeth may result in prolonged retention of primary or permanent dentition, diastemas, malposition of teeth and/or may cause psychological problems [3, 18, 19, 21]. In moderate and severe cases periodontal problems also occur, such as bleeding, enhanced risk of caries, and bone loss due to the excess of gingival tissue, presence of pseudopockets, and bacterial plaque accumulation. Sustained oral infections may ultimately impair systemic health due to their effect on the host immune system [3, 22, 23]. Another clinical problem found in patients with HGF is the recurrence of the lesions after surgical removal. In some cases, gingival overgrowth may reoccur within several weeks, months or even years after surgery, whereas in other patients the recurrence is not observed.. The patients with recurrent gingival overgrowth may also present some psychological issues due to frequent surgical interventions or postoperative complications [4, 20, 24–30]. Clinical manifestation, postoperative complications, and management of a highly recurrent, fibrotic gingival overgrowth have been well illustrated in a recently published clinical case report from our laboratory. In brief, an 11-year-old Caucasian female presented for evaluation of a gingival overgrowth that caused functional, hygienic, and psychological problems. Intraoral clinical examination revealed a painless, firm, and pale pink tissue covering 30% of her teeth crowns in the maxilla and the mandible. The recurrence of 25% of the pre-operative tissue volume in the maxilla and 45% of the initial tissue volume in the mandible was observed two and four weeks post-gingivectomy, respectively. Two months later, a gradual decrease in the recurrence was noted in both jaws. In contrast, the follow-up examinations at one, three, and 6 months after gingivectomy carried out in a 13-year**-**old sister of the patient revealed uneventful healing and no signs of recurrence, whereas complete surgery of diffuse overgrowth within mandibular and maxillary arches was performed in a 32-year-old mother of the patient resulted in the recurrence twelve months post-surgery, particularly in the mandible [18, 25]. These observations showed that the clinical appearance, episodes, and anatomical region of recurrence may vary among members of the same family and stand in accordance with other communications published over the last two decades, indicating that HGF represents a clinically heterogeneous entity. The phenotypic heterogeneity of HGF is presented in Fig. 1.

Besides an isolated form manifested in HGF, a fibrous gingival overgrowth can also occur as part of some genetic syndromes and diseases, causing serious disabilities e.g., mental deficiency and epilepsy in Ramon syndrome (MIM 266270) and Zimmermann-Laband syndrome (MIM 135500), progressive sensorineural hearing loss in Jones syndrome (MIM 135550), and many others [3, 31–36].

## Pathogenic variants of genes in HGF

**Fig. 2:**
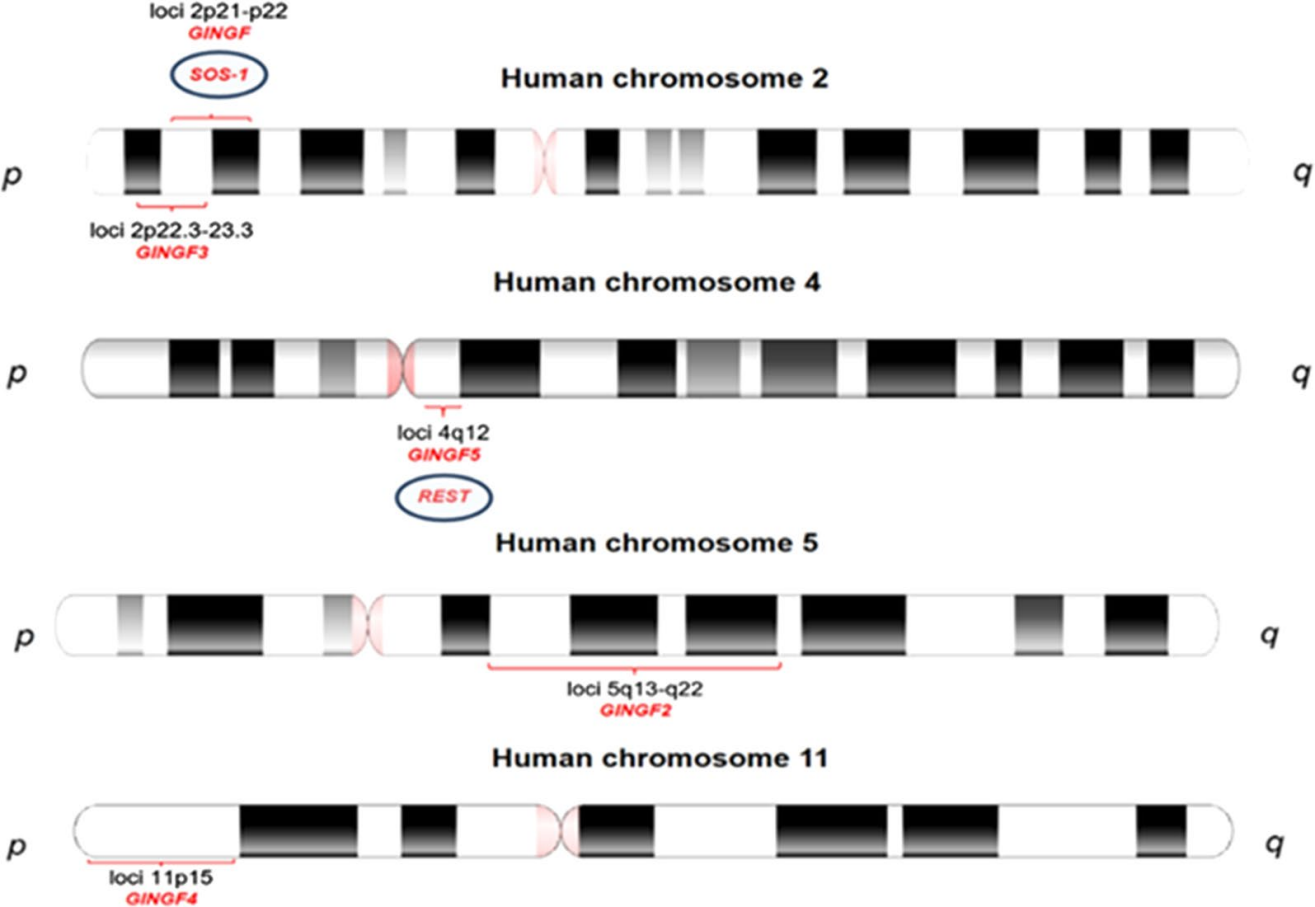
Schematic representation of the loci and affected genes associated with a non-syndromic variant of HGF. Two loci are present on chromosome 2 (GINGF, 2p21-p22 and GINGF3, 2p22.3-p23.3), one on chromosome 4 (GINGF5, 4q12), chromosome 5 (GINGF2, 5q13-q22) and one on chromosome 11 (GINGF4, 11p15). Pathogenic variants  of the *SOS-1* (Son**-**of**-**Sevenless**-**1) and *REST* (RE1-silencing transcription factor) genes were associated with GINGF and GINGF5, respectively

Non-syndromic HGF is transmitted as a Mendelian trait in an autosomal-dominant or, less frequently, an autosomal-recessive mode [1]. The incidence ratio of the condition does not depend on gender. Linkage analysis of families suffering from a non-syndromic autosomal-dominant variant of HGF revealed several regions on chromosomes that may potentially contain pathogenic variants of genes contributing to this condition. The candidate loci have been identified on chromosome 2p21-p22 (GINGF, HGF1, MIM 135300) in a Brazilian family, and chromosomes 5q13-q22 (GINGF2, MIM 605544), 2p22.3-p23.3 (GINGF3, MIM 609955), and 11p15 (GINGF4, MIM 611010) in several Chinese families [6, 7, 9, 10] (Table 1, Fig. 2).

**Table 1:** Chromosomal regions with affected or candidate genes contributing to non-syndromic HGF

Chromosomal region/gene locus	Affected^#^ or candidate^##^ gene	Gene/locus MIM number	Disease type	Phenotype MIM number	Inheritance	References
2p21-p22 (D2S1788, D2S441) GINGF, GINGF1, GGF1	*SOS-1*^#^	182530	Hereditary gingival fibromatosis type 1 (HGF1)	135300	AD	[6, 8, 12, 65]
2p16-p13 (D2S1788, D2S2298) GINGF	–	–	Hereditary gingival fibromatosis type 1 (HGF1)	135300	AD, AR	[64, 65]
5q13-q22 (D5S1491, D5S1453) GINGF2, GGF2	*CAMK4*^##^	114080	Hereditary gingival fibromatosis type 2 (HGF2)	605544	AD	[9, 66, 67]
2p23.3-p22.3 (D2S2221, D2S1788) GINGF3, GGF3	–	–	Hereditary gingival fibromatosis type 3 (HGF3)	609955	AD	[7, 68]
11p15 (D11S1984- D11S1338) GINGF4, GGF4	–	–	Hereditary gingival fibromatosis type 4 (HGF4)	611010	MI	[10, 69]
4q12 GINGF5, GGF5	*REST*^#^	600571	Hereditary gingival fibromatosis type 5 (HGF5)	617626	AD	[11, 70]

O(MIM), Online Mendelian Inheritance in Man; AD, autosomal dominant; AR, autosomal recessive; MI, maternal inheritance; *CAMK4*, calcium/calmodulin-dependent protein kinase IV; *SOS-1*, Son-of-Sevenless-1; *REST*, RE1**-**silencing transcription factor

^#^ affected gene, ^##^ candidate gene contributing to HGF

### Analysis of the *SOS-1* gene

Further sequencing analyses revealed an insertion of a single base (cytosine) in exon 21 (g.126,142-126,143insC; c.3248-3249insC) of the *SOS-1* gene (MIM 182530) in the 2p21-p22 candidate region, underlying GINGF locus which segregated in a dominant manner over four generations of a large Brazilian family with HGF. The chromosomal region 2p21-p22 has been discovered and described as the first candidate region containing the pathogenic variant, which contributes to HGF, hence the HGF induced by insertion of cytosine in exon 21 of the *SOS-1* gene has been designed as “type 1” (HGF1) [12]. Wild type human SOS-1, a bifunctional guanine nucleotide exchange factor (GEF) is a highly conserved 1,333-amino acid protein that is constitutively maintained in a down-regulated state under physiological conditions. The carboxyl-terminal domain of this protein exerts negative allosteric control on the interaction of the SOS-1 catalytic domain with Ras and Rac related proteins in cells. Ras regulates several protein kinases, including members of the mitogen-activated protein kinase (MAPK), phosphoinositide-3 kinase (PI3K), and proteins of the Rho family [37]. Indirect activation of Ras by SOS-1 resulting in phosphorylation of extracellular signal-regulated kinase 1/2 (ERK1/2) of the MAPK signaling pathway or leading to activation of downstream molecules involved in the PI3K pathway and the Ral nucleotide exchange factor (RalGDS) family are the key mechanisms regulating cell proliferation, survival, gene transcription, and differentiation [38]. SOS-1 also acts as a GEF for Rac when it forms a complex with the adaptor molecules E3b1 and Eps8. Activation of Rac regulates the actin remodeling and cytoskeletal organization that is critical for the transport of signaling molecules inside the cell, cell adhesion, and migration. Whether SOS-1 will function as a Ras or Rac exchange factor is dictated by its C-terminal proline-rich region, which contains binding sites for SRC Homology 3 (SH3) domains present in both adaptor proteins, growth factor receptor-bound protein 2 (Grb2), and E3b1 [39, 40]. Therefore, competition between these two adaptor proteins for binding to SOS-1 may determine whether the Ras or the Rac pathway gets activated [41, 42]. Phosphorylation of SOS-1 on tyrosine residues by the MAPK pathway does not affect the stability of the SOS-1/E3b1/Eps8 complex, but it disrupts the SOS-1/Grb2 complex [43]. Hence, phosphorylation of SOS-1 provides a mechanism to balance signaling between the Ras and Rac pathways. In addition to growth factor receptors, certain integrin-type extracellular matrix (ECM) receptors also regulate the Ras and Rac pathways through SOS-1 [44–46]. The single-cytosine insertion causes a frameshift and an early termination of the protein biosynthesis which yields a chimeric 1,105-amino acid protein that consists of 1,083 SOS-1 N-terminal amino acids followed by 22 replaced amino acids. Since the resultant chimeric protein lacks the regulatory carboxyl-terminal domain, it has augmented activity. This suggests that, in HGF1, mutated SOS-1 is constantly in an activated state, which may increase the activity of the MAPK pathway. The consequence is inappropriate transcriptional control of genes required for cell proliferation, differentiation, migration, ECM, and growth factors signaling [12]. Similar to humans, studies in several species, including yeast and *Drosophila melanogaster* indicate that truncation of the proline-rich carboxyl-terminal region of SOS-1 is associated with a gain-of-function [47]. Additionally, a transgene constructs with an SOS-1 carboxyl-terminal deletion (called “SOS-F”) induces skin tumor development in mice [48]. Furthermore, Lee et al. reported increased proliferation of fibroblasts and significantly increased levels of collagen in the histological studies of gingival tissue of the patients with HGF caused by the g.126,142-126,143insC pathogenic variant of the *SOS-1* [49]. Similarly, analyses in monolayer and three-dimensional cultures of gingival fibroblasts harboring the g.126,142-126,143insC in the *SOS-1* demonstrated increased cell proliferation rates, an altered ability to attach, an enhanced propensity to form protrusions with lamellipodia and dense staining of the ECM indicating clustering of collagen fibers. These data indicate that the presence of the g.126,142-126,143insC in exon 21 of the *SOS-1* gene producing the truncated protein chimera p.K1084fsX1105 induces HGF1 in an extended kindred through a gain-of-function mechanism [6, 12, 49]. Moreover, this pathogenic variant of the *SOS-1 * has been suggested to be associated with increased fibroblast proliferation and collagen matrix synthesis *in vivo* and *in vitro* [49].

On contrary, the analyses carried out in patients from two unrelated, Polish families with a history of non-syndromic autosomal-dominant HGF did not confirm single cytosine insertion (g.126,142-126,143insC) nor other pathogenic variants in exon 21 of the *SOS-1* gene. Further analyses of those families also did not reveal other insertions, any deletion, or substitution in the contiguous exons 19, 20, and 22 of the *SOS-1* [14]. As previously discussed, the single cytosine insertion in exon 21 of the *SOS-1* has been associated with increased synthesis of collagen type I and increased proliferation of gingival fibroblasts [49]. Although the patients from Polish families presented a highly fibrotic phenotype of HGF and increased proliferation rates of gingival fibroblasts, such associations were not found in our studies [14, 17, 50]. Likewise, Ma et al. did not detect a single-cytosine insertion in exon 21 nor any other pathogenic variant in 23 exons of the *SOS-1* gene in three multigeneration Chinese families with isolated HGF [13]. These observations suggest that the analyzed families from Poland and China are not affected by HGF1, which indirectly indicates that  the pathogenic variant(s) of other genes inducing other type(s) of HGF might be associated.

### Pathogenic variants of the *REST* gene

In fact, in a more recent study performed by Bayram et al., the WES analysis of eleven affected individuals with HGF from three unrelated families led to the identification of three heterozygous pathogenic variants predicted by conceptual translation to result in premature termination of the nascent transcript, two frameshifts, and one nonsense allele in the *REST* gene that has been mapped in the GINGF5 locus on chromosome 4 (GINGF5 on 4q12, MIM 617626) [11] (Table 1, Fig. 2).

Two different heterozygous truncating variants in the *REST* gene including a frameshift deletion (GenBank: NM_005612.4; exon 4; c.2865_2866delAA [p.Asn958Serfs*9]; chr4:57,797,888_CAA>C were identified in family 1 and a nonsense variant (GenBank: NM_005612.4; exon 4; c.1310T>A [p.Leu437*]; chr4:57,796,334_T>A was detected in family 2. Both affected siblings from family 1 were found to be heterozygous for the identified frameshift c.2865_2866delAA (p.Asn958Serfs*9) variant allele, the mother was found to be wild-type, while the mildly affected father also appeared to contain two wild-type alleles. However, the observed mild HGF phenotype in the father suggested possible tissue-specific mosaicism and transmission of the frameshift variant to the affected siblings from the father. In family 2, all five affected individuals, including the proband, two siblings, mother, and a maternal uncle were heterozygous carriers for the WES-identified stop-gain variant (c.1310T>A [p.Leu437*]) and the unaffected maternal aunt were wild-type at this locus. In family 3 the *de novo REST* variant (GenBank: NM_005612.4; exon 4; c.2413delC [p.Leu805Phefs*38]; chr4:57,797,436_CC>C) was identified in the proband. The *REST* gene is located on chromosome 4q12 and encodes a 1097-amino acid zinc finger protein. REST protein has a critical function as a transcriptional repressor during embryogenesis and neurogenesis and exerts an important role in several cellular mechanisms, such as tumor-suppressor as well as osteoblast, cardiac and hematopoietic differentiation [51–55]. The data from computational algorithm performed by Bayram et al. suggest that truncating variants may act through either a dominant-negative (antimorphic) or gain-of-function (neomorphic) effect, rather than by a haploinsufficiency mechanism, indicating that mutant transcripts may reduce the repressor function of REST in gingival fibroblasts from HGF-affected donors [11]. Although the pathophysiologic mechanisms underlying HGF remain not fully understood, it is considered that the pathologic manifestation of gingival overgrowth is driven by the excessive production of ECM components, particularly collagen type I by gingival fibroblasts [17, 18, 56–59]. Production of ECM is controlled by cytokines and growth factors that initiate and/or modulate signaling cascades mediated by specific receptors expressed by fibroblastic cells. The most potent cytokines involved in many biological processes, including regulation of collagen metabolism in connective tissues are transforming growth factor-β (TGF-β), connective tissue growth factor (CTGF), and IL-6 [58–60]. Kong et al. showed that putative REST target genes were widely involved in TGF-β signaling, whereas the inhibition of REST upregulated the TGF-β signaling pathway [61]. Another study implemented on neuroendocrine differentiation in prostate cancer cells revealed that knockdown of *REST* activates the IL-6-induced autophagy, while IL-6-induced neuronal cell morphology changes in prostate cancer cells were significantly inhibited due to the *REST* overexpression [62]. Taken together, it has been speculated that the truncating variants  of the *REST* gene may reduce the repressor function of REST that would induce overexpression of the *TGF-β* gene and upregulation of TGF-β signaling pathways, indirectly leading to increased synthesis and accumulation of collagen type I in HGF.

Bayram et al. also reported the availability of some truncating variants in the last exon of the *REST* gene in the public databases, i.e., ExAC [11]. However, since they did not have access to the samples and lack clinical information from that database, they could not validate these putative truncating variants nor check whether the individuals with truncating variants  were affected by HGF or not. Importantly, the calling of indels in genome-wide sequencing remains a challenge because of poor mappability [63]. Therefore, the frameshift variants identified in exome/genome sequencing need to be confirmed with DNA (Sanger) sequencing or allele-specific cloning.

### Overview of other candidate genes

Xiao et al. identified a four-generation Chinese family in which the gingival overgrowth manifests one year after birth, without the combined phenotypes of mental retardation, deafness, and hypertrichosis, thereby indicating non-syndromic HGF. The genome-wide search enabled to exclude the loci identified erenow for HGF and map the GINGF2 locus on the chromosome 5q13-q22 (MIM 605544) in this family. A strong candidate gene located within this region is calcium/calmodulin-dependent protein kinase IV (CAMK4) (MIM 114080) [9]. It is a multifunctional serine/threonine protein kinase, that mediates Ca^2+^-signaling pathways and is expressed in lymphocytes, neurons, male germ cells, and gingival tissue. It is, therefore hypothesized, that gingival overgrowth induced by cyclosporine A in genetically susceptible individuals may result from increased phosphorylation and activity of CAMK4 [9, 66, 67].

In the study of Hwang et al., instead of identification of specific pathogenic variants of genes responsible for HGF, the researchers proposed the candidate representative genes of the clusters of related genes potentially associated with non-syndromic HGF using WES followed by GSEA, and a protein functional network study [15]. A three-generation family whose grandparents and parents have normal gingiva while the children have gingival fibromatosis was enrolled in the study. A 13-year-old male showed gingival and alveolar overgrowth in the maxillary posterior area. His nine-year-old sister reported delayed eruption of teeth and was unable to close her mouth completely due to generalized gingival overgrowth. Her panoramic radiograph revealed delayed root development of primary second maxillary molars and mandibular canines. These two affected individuals had no history of systemic disease in pediatrics. Histological examinations of tissues obtained from the patients showed gingival fibrosis with myxoid changes. Protein functional network analysis was performed to see how genes from diverse functions were associated with each other in biological coordination and how the interactions between them might be associated with the pathogenesis of HGF. To better understand the function of genetic variants from HGF patients, the investigators searched for significantly enriched functional clusters. Among the top 10 clusters ranked by GSEA, multiple genes sets related to fibronectin, autoimmunity/immunity, C2 domain, myosin, microtubule, epidermal growth factor (EGF), GTPase, ATP binding, SH3 domain, and immunoglobulin were discovered [15]. In brief, ATP binding and fibronectin clusters were located in the center of a functional association network, which implies that fibronectin and ATP binding might be associated with the central cause of gingival hyperplasia. In turn, the immunoglobulin and EGF clusters were linked with fibronectin, while C2 domain, GTPase, myosin, and microtubule clusters were linked with ATP binding.

## Conclusions

Although the studies on the genetic background of HGF have been initiated in the late’90s, the pathogenic variants  inducing HGF were discovered only in the *SOS-1* and the *REST* genes. The *CAMK4* was identified as a strong candidate gene, while fibronectin and ATP binding clusters were found as candidate representatives of the clusters of related genes potentially associated with non-syndromic HGF. Altogether, these observations clearly indicate the genetic heterogeneity of non-syndromic HGF and point out the importance of further experimental as well as bioinformatic studies of the *SOS-1, REST,* and other genes located within previously reported candidate chromosomal regions as well as initially described candidate genes and/or associated clusters of genes, such as fibronectin, employing numerous unrelated multi-generation families probing to identify novel pathogenic variants of genes  associated with HGF. This strategy will help to unravel the molecular  mechanisms underlying gingival overgrowth, followed by the identification of specific targets for less invasive therapies into the dental practice.

## Data Availability

Data sharing is not applicable to this article, as no datasets were generated or analyzed during the current study. All articles reviewed for this study are mentioned in this article.

## Abbreviations

AD: Autosomal dominant
AR: Autosomal recessive
*CAMK4*: Calcium/calmodulin-dependent protein kinase IV
CTGF: Connective tissue growth factor
ECM: Extracellular matrix
EGF: Epidermal growth factor
ERK1/2: Extracellular signal**-**regulated kinase 1/2
GEF: Guanine nucleotide exchange factor
GF: Gingival fibromatosis
Grb2: Growth factor receptor**-**bound protein 2
GSEA: Gene set enrichment analysis
HGF: Hereditary gingival fibromatosis
HGF1: Non-syndromic hereditary gingival fibromatosis type 1
MAPK: Mitogen**-**activated protein kinase
MI: Maternal inheritance
O(MIM): Online Mendelian Inheritance in Man
PI3K: Phosphoinositide**-**3 kinase
RalGDS: Ral nucleotide exchange factor
*REST*: RE1**-**silencing transcription factor
SH3: SRC Homology 3 domains
*SOS-1*: Son**-**of**-**Sevenless-1
TGF**-**β: Transforming growth factor-β
WES: Whole-exome sequencing
